# Impact of COVID-19 Related Maternal Stress on Fetal Brain Development: A Multimodal MRI Study

**DOI:** 10.3390/jcm11226635

**Published:** 2022-11-09

**Authors:** Vidya Rajagopalan, William T. Reynolds, Jeremy Zepeda, Jeraldine Lopez, Skorn Ponrartana, John Wood, Rafael Ceschin, Ashok Panigrahy

**Affiliations:** 1 Department of Radiology, Children’s Hospital Los Angeles, Keck School of Medicine University of Southern California, Los Angeles, CA 90033, USA; 2 Department of Biomedical Informatics, University of Pittsburgh, Pittsburgh, PA 15206, USA; 3 Department of Radiology, Children’s Hospital Los Angeles, Los Angeles, CA 90027, USA; 4 Neuropsychology Core, The Saban Research Institute, Children’s Hospital Los Angeles, Los Angeles, CA 90027, USA; 5 Department of Pediatric Radiology, Keck School of Medicine University of Southern California, Los Angeles, CA 90033, USA; 6 Departments of Radiology and Pediatrics, Children’s Hospital Los Angeles, Keck School of Medicine University of Southern California, Los Angeles, CA 90033, USA; 7 Department of Radiology, University of Pittsburgh School of Medicine, Pittsburgh, PA 15206, USA; 8 Department of Pediatric Radiology, Children’s Hospital of Pittsburgh of UPMC, Pittsburgh, PA 15224, USA

**Keywords:** fetal brain function, maternal stress, COVID-19 pandemic

## Abstract

Background: Disruptions in perinatal care and support due to the COVID-19 pandemic was an unprecedented but significant stressor among pregnant women. Various neurostructural differences have been re-ported among fetuses and infants born during the pandemic compared to pre-pandemic counterparts. The relationship between maternal stress due to pandemic related disruptions and fetal brain is yet unexamined. Methods: Pregnant participants with healthy pregnancies were prospectively recruited in 2020–2022 in the greater Los Angeles Area. Participants completed multiple self-report assessments for experiences of pandemic related disruptions, perceived stress, and coping behaviors and underwent fetal MRI. Maternal perceived stress exposures were correlated with quantitative multimodal MRI measures of fetal brain development using multivariate models. Results: Increased maternal perception of pandemic related stress positively correlated with normalized fetal brainstem volume (suggesting accelerated brainstem maturation). In contrast, increased maternal perception of pandemic related stress correlated with reduced global fetal brain temporal functional variance (suggesting reduced functional connectivity). Conclusions: We report alterations in fetal brainstem structure and global functional fetal brain activity associated with increased maternal stress due to pandemic related disruptions, suggesting altered fetal programming. Long term follow-up studies are required to better understand the sequalae of these early multi-modal brain disruptions among infants born during the COVID-19 pandemic.

## 1. Introduction

The COVID-19 pandemic created many, unprecedented disruptions to everyday life particularly in 2020–2022 before vaccines were widespread. In addition to disruptions around employment, childcare, housing, and nutrition, pregnant women also suffered negative experiences related to support and care during pregnancy and childbirth. Social isolation, reduced access to child and elder care, COVID-19 infection risk, and changes to medical policies around pre and postpartum care were reported to be the most common stressors among pregnant women [1,2]. Pregnant women are particularly vulnerable to mood and anxiety related disorders [3] which are exacerbated during natural disasters or stressful events [4,5]. Unsurprisingly, pregnant women indicated elevated levels of stress during the COVID-19 pandemic [6]. In addition to health consequences for the mother, increased maternal stress has an intergenerational impact on fetal development [7,8]. Increased maternal stress during pregnancy is known to alter the fetal brain and adversely impact postnatal neurodevelopmental outcomes [9,10,11,12].

Studies of infants born during the COVID-19 pandemic have reported reduced cognitive, motor, and emotional development compared to those born pre-pandemic [7,8], with increased prenatal stress directly associated with adverse affect and temperament [13,14]. Simultaneously, changes to brain structure and function have also been reported in infants born during the pandemic [15]. Lu et al. [16] reported volumetric reductions in the brain among fetuses of women pregnant during the pandemic compared to a pre-pandemic cohort. Their findings showed a negative relationship between general maternal stress and fetal brain volumes. However, their cohort did not show an increase in maternal stress or anxiety during a pandemic, and they did not measure maternal stress or anxiety specifically linked to the pandemic. Additionally, there is no data on if or how emerging functional networks in the fetal brain, which are known to be sensitive to ma-ternal stress, were impacted by pandemic related maternal stress. Early aberrations to functional organization of the brain are well known to have deleterious downstream effects in brain and behavioral development. As such, a multimodal imaging study is important to better understand how prenatal maternal stress sets up the offspring’s brain for a trajectory of compounding aberrant development.

Understanding the impact of pandemic related maternal stress on fetal development allows us to identify risk and resilience factors to mitigate maternal stress and consequently minimize the intergenerational effect of pandemic related stress. Coping behaviors, in response to stressful events, are known to be modifiable targets to mitigate maternal stress and anxiety [17,18]. Given the extraordinary nature of pandemic related stressors, there is little information on various coping behaviors that pregnant women have adopted during the pandemic [19,20,21]. Despite its observational nature, information on coping behaviors to pandemic related stressors allow clinical care teams to design and implement support programs aimed at improving maternal mental health during pregnancy and child outcomes.

In this work, we investigated the impact of maternal stress due to pandemic related disruptions in pregnancy support and care on structural and functional development of the human fetal brain. Our primary hypothesis is that increased maternal stress would predict quantitative alterations in structural and functional characteristics of the fetal brain. Secondarily, we compared coping behaviors between pregnant women reporting high vs. low levels of pandemic related stress.

## 2. Materials and Methods

### 2.1. Subject Demographics

Pregnant mothers, living in the greater Los Angeles area were recruited using flyers, social media ads, and referrals from community partner clinics at Children’s Hospital Los Angeles (CHLA) from November 2020–November 2021. Enrollment eligibility included healthy, pregnant women between 18–45 years with singleton, uncomplicated pregnancies (confirmed by ultrasound) between 21–38 gestational weeks (GW). Exclusion criteria were multiple gestation, fetal or genetic anomalies, congenital infection, and maternal contraindication to MRI. Informed consent for the study was obtained under a protocol approved by the Institutional Review Board at CHLA. Demographics, perinatal health history, and self-assessment surveys of consented participants were gathered via online survey within 24 h prior to MRI.

### 2.2. Stress and Coping Behavioral Assessments

Participants were asked to complete the Coronavirus Perinatal Experiences-Impact Survey [22] (COPE-IS). This is a self-assessment questionnaire, available in multiple languages, to assess feelings and experiences of pregnant women and new mothers in relation to disruptions caused by the COVID-19 pandemic. Questions in this assessment were adapted from multiple validated questionnaires such as the Brief Symptom Inventory [23] PTSD checklist from DSM-5 [24], and the Johns Hopkins Mental Health Working Group. In this study, we only included questions pertinent to the prenatal period. Perceived maternal stress was computed as described here [21,22] and will be referred to as COPE-Stress going forward. Participants also completed the Brief COPE questionnaire [25], which is an abbreviated form of the COPE (Coping Orientation to Problems Exposed) questionnaire [26]. This is a self-assessment of a wide range of coping behaviors including both maladaptive coping (includes substance use, venting, behavioral disengagement, denial, self-blame, and self-distraction) [27] and adaptive coping (includes humor, planning and seeking social support, use of emotional and instrumental support, positive reframing, religion, and acceptance) [26,28]. This questionnaire has been validated in multiple languages and cultural contexts to be correlated to perceived stress and mental well-being.

### 2.3. Child Opportunity Index (COI)

Neighborhood socio-economic environment (SEE) is a known modifier of overall maternal stress during pregnancy [29], pandemic related stress [30], and offspring outcomes [31]. Family income is often used to measure SEE. However, the quality of life associated with absolute income number varies regionally based on cost of living, social policies, environmental factors, etc. To overcome these limitations, we chose to represent SEE using childhood opportunity index (COI). COI is a multi-dimensional, nationally normed measure of the quality of social, environmental, health, and educational resources available at each zip code [32]. We extracted maternal COI using self-reported zip code at the time of the MRI visit and will be referred to as COI-SEE going forward.

### 2.4. Image Acquisition

Pregnant mothers were prospectively recruited between 24–38 GW and imaged on 3.0 T Philips Ingenia scanner (Philips Healthcare, Best, The Netherlands). Multiplanar single-shot turbo spin echo imaging was per-formed (TE = 160 ms, TR = 9000–12,000 ms, 3 mm slice thickness, no interslice gap, 1 × 1 mm in plane resolution). Fetal brains were scanned in each of three planes for three times resulting in nine images per subject and images were repeated if excessive motion was present. Echo-planar imaging (EPI) BOLD images were also collected with the following parameters: FOV = 300 mm TR = 2000 ms, TE = 31–35 ms (set to shortest), flip angle = 80°, with an in-plane resolution of 3 × 3 mm^2^, slice thickness of 3.0 mm and 0.0 mm intra-slice gap. 150 timepoints were recorded for each BOLD image and two images were collected for each subject.

### 2.5. Image Processing

#### 2.5.1. Brain Structure

All structural brain images were verified as being typical for gestational age by a board certified neuroradiologist (SP). For each subject, various 2D stacks of the T2 images were visually assessed to identify and discard stacks with large, spontaneous fetal motion. In each stack, the fetal brain was localized from surrounding tissue. For each subject, multiple 2D stacks were motion corrected and reconstructed, using a slice-to-volume reconstruction [33] into a 3D volumetric T2 image with an isotropic resolution of 1 mm^3^. Reconstructed fetal brains were processed through a bespoke, automated fetal segmentation pipeline. Each fetal brain was normalized (affine followed by non-rigid) to a probabilistic atlas [34] of equivalent gestational age using Advanced Normalization tools [35]. Segmentations were manually inspected for accuracy and subjects with failed segmentations were discarded. The resulting segmentation maps were subsequently refined. To ensure consistency across different gestational ages, transient structures only present in the tissue atlas from 21–30 weeks of gestation such as the subplate, intermediate zone, and ventricular zone were combined with the corpus collosum and labeled as developing WM (WM). Cerebrospinal fluid (CSF) segmentation was refined as intra-ventricular (within lateral ventricles) and extra-axial CSF. Due to the small size and relative difficulty in segmenting the hippocampus and amygdala, both structures were combined into a hippocampus-amygdala complex. Deep grey tissue was defined as the combination of the caudate, putamen, thalamus, fornix, internal capsule, subthalamic nucleus, and hippocampal commissure. Right and left hemispheric labels were combined into a single volume for each structure. The final segmentation yielded volumes of the following structures: cortical plate, developing white matter, intra-ventricular CSF, extra-axial CSF, deep gray tis-sues, cerebellum, hippocampal-amygdala complex, and brainstem. A total brain volume (TBV) was generated for each subject as the sum of all tissues.

#### 2.5.2. Brain Function

BOLD imaging of the fetal brain is prone to spontaneous fetal motion which is com-pounded by lower signal to noise ratio and spatial resolution. While modern motion correction algorithms effectively attenuate the effects of subject motion on the temporal data, they are limited in effect beyond small degrees of motion. Any robust voxel-wise approach to functional fetal imaging would yield a prohibitively low number of subjects with usable data. We therefore chose to implement a whole-brain temporal signal approach to fetal functional imaging. Resting state images were first motion corrected using FSL’s MCFLIRT routine, using the first frame as the registration target, and a mean framewise displacement threshold >0.2 mm to eliminate frames with excessive motion. As the intent of this study was to use minimally processed data using framewise measures, as opposed to voxel wise measures, we made no prior assumptions on physiological or nuisance frequency thresholds in fetal functional imaging and did not apply any bandpass filtering. A mean brain signal image was then generated by averaging across every frame in the sequence. This mean signal image was used as the source image for brain extraction to generate a brain mask. Brain extraction was done by using an adaptive routine that iterated between using FSL’s Brain Extraction Tool (BET) [36] and AFNI’s Skullstrip, using decreasingly smaller thresholds for brain tissue [37]. This approach yielded a good approximation of the fetal brain, with a minimal manual correction step required for final brain masking. The brain mask was then propagated across each frame in the temporal sequence to extract only fetal brain voxels.

Using the mask generated above, we averaged the whole brain BOLD signal in each frame and generated statistical measures across time. The measures generated were temporal mean (average of the mean signal across frames), temporal variability (average of the standard deviation of the signal across frames), variance of the mean (variance of the mean signal in each frame), kurtosis of the mean (kurtosis of the mean signal in each frame). Finally, to assess for any signal or physiological drift, we calculated the autocorrelation of the mean signal in each frame, and the kurtosis and autocorrelation of the normalized signal across frames.

### 2.6. Statistical Analysis

#### 2.6.1. Brain Structure

Regression analysis was performed in Python (3.7) using the Statsmodel.api v0.13.2. We used multiple, linear regression to model the relationship of COPE-Stress Score, COI-SEE, and their interaction on TBV after adjusting for gestational age at MRI. Nested models of the covariates without interaction were also evaluated. Models were deemed to be significant if one or more of the covariates were statistically significant, and models including the interaction term were only selected over the simpler counterpart if they had a higher explained variance (R-squared) and/or lower Bayes’ Information Criteria (BIC). Using similar regression models, we individually assessed the relationship of COPE-Stress score and COI-SEE for each tissue volume listed in Section 2.5.1 (as a dependent variable). Secondarily, we also evaluated the relationship of COPE-Stress score and COI-SEE on tissue volumes normalized by TBV after adjusting for gestational age.

#### 2.6.2. Brain Function

Statistical analysis for brain functional metrics was similar to Section 2.5.1. A separate regression model was evaluated for each, individual functional metric (Section 2.5.2) with COPE Stress, COI-SEE, and their interaction as predictor variables after accounting for GA at MRI.

#### 2.6.3. Comparison of Coping Behaviors

Coping behaviors, both the Brief-COPE and COVID specific, were analyzed for differences between low and high stress mothers. Mothers were split into low, medium, and high stress categories based on tertiles of COVID Stress scores. Using Fischer Exact test, we compared if mothers reporting low and high stress used each coping behavior at significantly different amounts.

## 3. Results

### 3.1. Subject Demographics

Pregnant mothers were recruited prospectively for this study with a total of 45 mother-fetus dyads completed the MR imaging session. Three subjects had missing zip code information, and which resulted in missing COI-SEE data and was thus excluded from any analysis. After imaging, three subjects failed brain segmentation resulting in 39 subjects for structural regression results. A total of 43 subjects of the original 45 subjects had analyzable BOLD imaging and were used for the functional regression results (Table 1).

### 3.2. Brain Structure

There were no significant associations between absolute volumes of the various brain structures and perceived maternal stress, COI-SEE, or their interaction (Table 2). However, there was a significant positive association between normalized brain stem volume and perceived maternal stress (*p* = 0.03) but not with COI-SEE and the interaction of COI-SEE and maternal stress (Table 3) There were no significant associations between normalized volumes of other structures with COPE-Stress or COI-SEE.

### 3.3. Brain Function

Lack of significant relationship between autocorrelation metrics and the predictor variable confirmed the absence of any systematic signal or physiological drifts. We found a significant negative relationship between temporal variability and COPE Stress (*p* < 0.028) (Table 4). The temporal variability model including the interaction term between Cope Stress Score and COI SES had a slightly improved R-squared (0.267) but lower BIC and reduced statistical significance of the covariates, likely due to co-linearity. We there-fore report the original model without the interaction term. We found no other statistically significant relationships between fetal brain functional characteristics with COPE Stress or COI SEE.

### 3.4. Comparison of Coping Behaviors

**Figure 1:**
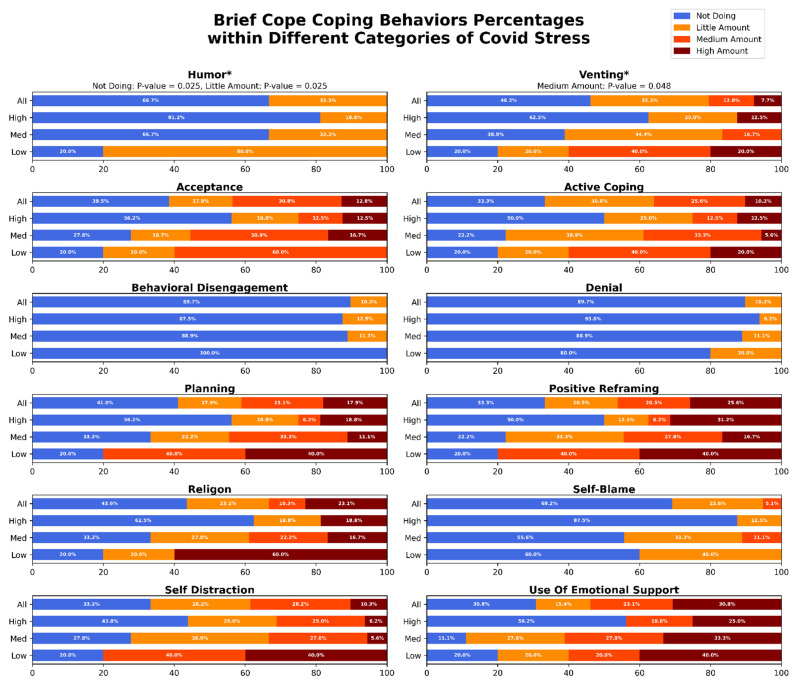
Prevalence of use of various subscales of coping behaviors within pregnant women in the study. The first row for each subscale represents the entire cohort. The second, third, and fourth rows correspond to prevalence of coping behaviors in participants reporting high, medium, and low COVID-19 related stress. * Denotes subscales with significantly different prevalence of use between mothers reporting high and low stress levels.

**Figure 2:**
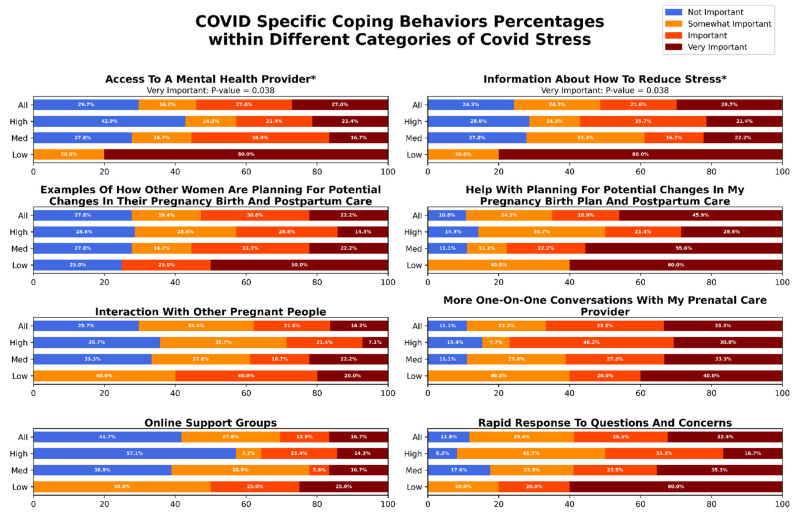
Self-reported importance of potential resources for management of stress associated with COVID-19 related disruptions among pregnant women in the study. The first row for each subscale represents the entire cohort. The second, third, and fourth rows correspond to prevalence of coping behaviors in participants reporting high, medium, and low COVID-19 related stress. * Denotes subscales with significantly different prevalence of use between mothers reporting high and low stress levels.

We compared coping behaviors between participants reporting high and low stress in our cohort. Figure 1 shows the prevalence of use of various coping behaviors, reported as percentage of total, among the participant in the study. Humor (*p*-value = 0.025) and venting (*p*-value = 0.048) were used more commonly by participants reporting low stress compared to those reporting high stress (Figure 1). We observed differential patterns of coping behavior use among mothers who reported high and low stress. Figure 2 shows, self-reported importance levels of potential resources for management of stress associated with COVID-19 related disruptions among pregnant women in the study. Access to a mental health provider (*p*-value = 0.038), and information about how to reduce stress (*p*-value = 0.038) were chosen as being ‘Very Important’ to women reporting low stress at a high amount than in women reporting high stress (Figure 2). No other behaviors were found to be significantly different between high and low stress mothers. A full summary of the results can be seen in Figure 1 and Figure 2.

## 4. Discussion

Our findings show that perceived maternal stress, in the setting of COVID-19 related care disruptions, impacts with structural and functional developmental of the fetal brain. Higher maternal stress was associated with increased brainstem volume (suggesting accelerated brainstem maturation) and globally decreased temporal variability of function (suggesting reduced functional connectivity) in the fetal brain. Additionally, we also found differences in the prevalence of specific coping behaviors between pregnant women who reported high stress compared to those who reported low stress. Our study is novel in the following aspects: (1) use multi-modal imaging to characterize fetal brain developmental changes due to maternal stress during COVID, and (2) characterization of adaptive coping behaviors which may provide resilience during this period of increased maternal stress.

We found that increased levels of maternal stress correlated with increased normalized brainstem volume suggesting relatively increased acceleration of brainstem maturation relative to cortical/supratentorial cerebral regions. Importantly, these results are consistent with prior studies that have correlated prenatal maternal stress and neonatal brainstem auditory evoked potentials (the speed at which the brainstem auditory evoked potential is conducted through the auditory nerve serves as a proxy for greater neural maturation) [38,39]. These studies have found significant relations between higher maternal prenatal distress and faster conductance, suggesting that greater maternal prenatal stress is associated with accelerated subcortical/brainstem neural maturation in neonates [40]. Our results are also consistent with the recent study by De Asis-Cruz et al. [41] which found that altered functional connectivity between brainstem and sensorimotor regionals were associated with high maternal anxiety scores.

We found that higher perceived maternal stress was associated with lower temporal variability in the fetal brain suggesting aberrations to foundational characteristics of connectivity and organization of emerging brain networks [42]. It has been well-established that perturbations to early brain connectivity architecture, during the critical fetal period, has long-standing effects on behavioral and psychiatric development among these children [43,44,45]. Altered temporal variability of brain BOLD are known to associated with adverse neurocognitive functioning of the brain [46,47,48]. Our findings of altered brain connectivity agree with previous findings of altered brain connectivity in infants of mothers who reported higher stress during the pandemic [15]. Behavioral and functional deficits particularly in the motor, cognitive and temperamental domain have been widely reported in various studies investigating the impact of maternal stress during the pandemic on child outcomes [7,8,13,14]. Increased maternal stress and anxiety traits (outside the setting of the pandemic) have been shown to alter functional architecture of the fetal brain [49]. Collectively, our and prior findings suggest that in utero alterations to brain architecture, associated with maternal stress during the pandemic, could underlie developmental deficits reported in these children. Further meta studies are needed to investigate the trajectory of brain development in children conceived and born during the pandemic.

Our findings suggest key differences in coping behaviors between pregnant women who reported low and high stress. Increased use of adaptive coping behaviors (particularly humor and venting) was more common among pregnant women who reported lower stress compared to those who reported higher stress. This association between in-creased use of adaptive, active coping and lower stress perception was reported across multiple studies of mental health in peripartum women during COVID-19 pandemic [21,50,51]. Our findings are also in agreement with generalized findings of positive relationship between active coping behaviors and improved mental well-being in pregnant women [52]. In questions regarding COVID-19 specific coping behaviors, pregnant mothers reporting low stress endorsed access to mental health information and providers as being key to wellness. Routine screening for prenatal stress, provision of stress management information, and improved access to prenatal mental health care provide potential avenues for improving mental health and associated outcomes in pregnant women regardless of pandemic conditions.

This study’s limitations include small sample size and recruitment limited to a single geographical area in the USA during the pandemic. Since the greater Los Angeles area was disproportionately affected by pandemic related disruptions, comparison to a multi-site cohort will provide greater statistical power thereby increasing the generalizability of our findings. The cross-sectional nature of prenatal stress assessment limits our ability to associate time-varying stress levels and fetal outcomes. However, all participating women became pregnant after pandemic-related restrictions were put in place. Lack of a pre-pandemic cohort limits our ability to pin-point if the differences in coping behaviors between pregnant women reporting low and high stress are specific adaptations to stress experienced during the pandemic. Consistent with the demographics of Los Angeles County, over 50% of the pregnant women in our study cohort identified as Hispanic. Cultural norms around coping behaviors, care and family support during pregnancy or postpartum periods should be factored into the interpretation of our findings. Future work could examine if disparities in healthcare utilization have been altered during the pandemic among pregnant women from diverse backgrounds [53,54].

## 5. Conclusions

Here, we reported the first multi-modal study of the impact of COVID-19 pandemic related maternal stress on fetal brain development. Our findings showed that increased maternal stress due to pandemic related disruptions was associated with structural and functional disruptions to fetal brain development and is suggestive of altered fetal programming. Comparing coping behaviors between pregnant women reporting higher and lower stress, our study provides insight into potential avenues for improved stress management and mental health outcomes among pregnant women.

## Figures and Tables

**Table 1:** Study participants’ demographic information.

Characteristic	Total	Range/Percentage of Total
**Total Participants**	45	
**Sex of fetus**	18	
Female	18	40%
Male	20	44.5%
Unknown	7	15.5%
	45	
**Total MRI’s**		
**GA, median (range), wk.**		
At MRI	31.57	(22.57 to 38.42)
At Birth	39.14	(33 to 41.86)
**Maternal age at MRI, median, yr.**	32	(18 to 43)
**Maternal parity**		
Primiparous	18	40%
Multiparous	22	49%
Unknown	5	11%
**Infant Weight, median, kg**	3.54	
Caucasian	8	18%
Hispanic or Latino	28	62%
Asian/Pacific Islander	7	16%
African American	1	2%
Middle Eastern	0	
Other or unknown	1	2%

**Table 2:** Raw brain structure volumes relationship to COVID stress and COI-SEE.

Volume (cm^3^)	COVID Stress Score		COI Nationally Normed Value		COI Stress Interaction	
	β (CI)	*p*-Value	β (CI)	*p*-Value	β (CI)	*p*-Value
Brainstem	3.89 × 10^0^, (−7.62 × 10^1^, 8.40 × 10^1^)	0.97	−2.81 × 10^−1^, (−1.41 × 10^1^, 1.35 × 10^1^)	0.99	4.06 × 10^−1^, (−1.18 × 10^0^, 1.99 × 10^0^)	0.86
Cerebellum	1.54 × 10^2^, (−4.84 × 10^1^, 3.56 × 10^2^)	0.61	3.28 × 10^1^, (4.13 × 10^−1^, 6.52 × 10^1^)	0.49	−1.95 × 10^0^, (−5.79 × 10^0^, 1.89 × 10^0^)	0.73
Cortical Plate	−7.33 × 10^2^, (−1.35 × 10^3^, −1.18 × 10^2^)	0.42	−3.78 × 10^0^, (−1.59 × 10^2^, 1.52 × 10^2^)	0.99	1.23 × 10^1^, (−1.43 × 10^0^, 2.60 × 10^1^)	0.55
Deep Grey	1.93 × 10^1^, (−1.83 × 10^2^, 2.22 × 10^2^)	0.95	2.76 × 10^0^, (−3.17 × 10^1^, 3.73 × 10^1^)	0.96	1.65 × 10^0^, (−2.28 × 10^0^, 5.58 × 10^0^)	0.78
Extra Axial CSF	−7.29 × 10^2^, (−1.74 × 10^3^, 2.81 × 10^2^)	0.63	−9.28 × 10^1^, (−3.06 × 10^2^, 1.21 × 10^2^)	0.77	1.76 × 10^1^, (−5.28 × 10^0^, 4.04 × 10^1^)	0.60
Hippocampus amygdala complex	−1.31 × 10^0^, (−2.72 × 10^1^, 2.46 × 10^1^)	0.97	−7.62 × 10^−1^, (−6.21 × 10^0^, 4.69 × 10^0^)	0.92	2.17 × 10^−1^, (−3.33 × 10^−1^, 7.68 × 10^−1^)	0.79
Intra ventricular CSF	2.59 × 10^1^, (−7.98 × 10^1^, 1.32 × 10^2^)	0.87	1.23 × 10^1^, (−1.15 × 10^1^, 3.60 × 10^1^)	0.73	−5.07 × 10^−1^, (−2.81 × 10^0^, 1.79 × 10^0^)	0.88
White Matter	−5.17 × 10^2^, (−1.69 × 10^3^, 6.58 × 10^2^)	0.77	−8.47 × 10^1^, (−2.99 × 10^2^, 1.30 × 10^2^)	0.79	1.19 × 10^1^, (−1.25 × 10^1^, 3.63 × 10^1^)	0.74
Total Brain Volume	−2.51 × 10^3^, (−6.81 × 10^3^, 1.80 × 10^3^)	0.69	−2.27 × 10^2^, (−1.05 × 10^3^, 5.96 × 10^2^)	0.85	5.92 × 10^1^, (−3.03 × 10^1^, 1.49 × 10^2^)	0.66

**Table 3:** Brain structure volumes’, after normalization to total brain volume, relationship to COVID stress, COI-SEE, and their interaction. * denotes statistically significant relationships.

Volume Normalized by Total Brain Volume	COVID Stress		Overall COI by Zip Code		COVID Stress and COI Interaction	
	β (CI)	*p*-Value	β (CI)	*p*-Value	β (CI)	*p*-Value
Brainstem	1.30 × 10^−4^, (9.00 × 10^−5^, 1.70 × 10^−4^)	0.03 *	1.00 × 10^−5^, (0.00 × 10^0^, 2.00 × 10^−5^)	0.65	0.00 × 10^0^, (0.00 × 10^0^, 0.00 × 10^0^)	0.31
Cerebellum	3.40 × 10^−4^, (1.50 × 10^−4^, 5.40 × 10^−4^)	0.24	7.00 × 10^−5^, (4.00 × 10^−5^, 1.10 × 10^−4^)	0.12	−1.00 × 10^−5^, (−1.00 × 10^−5^, 0.00 × 10^0^)	0.26
Cortical Plate	−1.42 × 10^−3^, (−2.10 × 10^−3^, −7.40 × 10^−4^)	0.16	−1.00 × 10^−5^, (−2.20 × 10^−4^, 2.00 × 10^−4^)	0.97	1.00 × 10^−5^, (−1.00 × 10^−5^, 3.00 × 10^−5^)	0.64
Deep Grey	1.90 × 10^−4^, (3.00 × 10^−5^, 3.60 × 10^−4^)	0.42	2.00 × 10^−5^, (−3.00 × 10^−5^, 6.00 × 10^−5^)	0.82	0.00 × 10^0^, (0.00 × 10^0^, 1.00 × 10^−5^)	0.90
Extra Axial CSF	1.10 × 10^−4^, (−7.00 × 10^−5^, 2.80 × 10^−4^)	0.68	−5.00 × 10^−5^, (−1.20 × 10^−4^, 2.00 × 10^−5^)	0.61	0.00 × 10^0^, (0.00 × 10^0^, 1.00 × 10^−5^)	0.60
Hippocampus amygdala complex	4.00 × 10^−5^, (2.00 × 10^−5^, 6.00 × 10^−5^)	0.22	0.00 × 10^0^, (−1.00 × 10^−5^, 1.00 × 10^−5^)	0.94	0.00 × 10^0^, (0.00 × 10^0^, 0.00 × 10^0^)	0.99
Intra ventricular CSF	1.20 × 10^−4^, (−4.00 × 10^−5^, 2.80 × 10^−4^)	0.61	5.00 × 10^−5^, (−1.00 × 10^−5^, 1.00 × 10^−4^)	0.55	0.00 × 10^0^, (−1.00 × 10^−5^, 0.00 × 10^0^)	0.64
White Matter	3.80 × 10^−4^, (−1.60 × 10^−4^, 9.20 × 10^−4^)	0.63	−3.00 × 10^−5^, (−1.80 × 10^−4^, 1.20 × 10^−4^)	0.89	−1.00 × 10^−5^, (−3.00 × 10^−5^, 0.00 × 10^0^)	0.62

**Table 4:** Brain functional metrics’ relationship to COVID stress and COI-SEE using linear modeling. * denotes statistically significant relationships.

	COVID Stress		Overall COI by Zip Code	
	β (CI)	*p*-Value	β (CI)	*p*-Value
Temporal mean of BOLD Signal	135.369, (−509.52, 38.1)	0.09	316.9634, (−604.97, 1238.9)	0.49
Temporal variability of BOLD Signal	−113.94, (−215.18, −12. 71)	0.03 *	−19.5173, (−360.388, 321.354)	0.91
Variance of framewise mean BOLD signal	−5336.81, (−2.87 × 10^4^, 1.81 × 10^4^)	0.65	−5191.57, (−8.4 × 10^4^, 7.36 × 10^4^)	0.9
Kurtosis of framewise mean BOLD signal	0.329, (−0.144, 0.802)	0.17	0.457, (−1.135, 2.049)	0.57
Autocorrelation of framewise mean BOLD	−6.828 × 10^6^, (−1.41 × 10^7^, 4.89 × 10^5^)	0.07	1.005 × 10^7^, (−1.46 × 10^7^, 3.47 × 10^7^)	0.41

## Data Availability

Due to limitations of informed consent, data from the study cannot be shared. However, Methodologies and techniques from the study will be made available via direct email to the corresponding author.
